# Functional Responses of the Warehouse Pirate Bug *Xylocoris flavipes* (Reuter) (Hemiptera: Anthocoridae) on a Diet of *Liposcelis decolor* (Pearman) (Psocodea: Liposcelididae)

**DOI:** 10.3390/insects16010101

**Published:** 2025-01-19

**Authors:** Augustine Bosomtwe, George Opit, Kristopher Giles, Brad Kard, Carla Goad

**Affiliations:** 1 Department of Entomology and Plant Pathology, Oklahoma State University, 127 Noble Research Center, Stillwater, OK 74078, USA; george.opit@okstate.edu (G.O.); kris.giles@okstate.edu (K.G.); b.kard@okstate.edu (B.K.); 2 CSIR-Plant Genetic Resources Research Institute, Bunso P.O. Box 7, Ghana; 3 Department of Statistics, Oklahoma State University, 301 Mathematics, Statistics and Computer Sciences, Stillwater, OK 74078, USA; carla.goad@okstate.edu

**Keywords:** biological control, predator, bio-based IPM, psocid, stored-product pest

## Abstract

Psocids are difficult to manage using grain protectants and phosphine hence research on non-chemical control methods. This study evaluated the effectiveness of *Xylocoris flavipes* (Reuter) (Hemiptera: Anthocoridae) at managing *Liposcelis decolor* (Pearman) (Psocodea: Liposcelididae). The functional responses of adult♀ and nymphs of *X. flavipes* on a diet of nymphs, adult♂, and adult♀ of *L. decolor* were determined under laboratory conditions at 28 ± 1 °C, 63 ± 5% RH, and a 0:24 (L:D) photoperiod. Maximum likelihood estimates (MLEs) of a logistic regression analysis showed that the functional responses of the life stages of *X. flavipes* on diets of three stages of *L. decolor* were Holling Type II. Estimates of the attack rate (*a*), handling time (*Th*), maximum predation (*K*) per day, and predation efficiency (*η*) per day for the two predator life stages against each prey life stage showed that the adult♀ *X. flavipes* predation rate was better compared with the nymphs. The adult♀ *X. flavipes* per capita consumption rate and searching efficiency were also higher than those of the nymphs. The data showed adults of *L. decolor* are more favorable for adult♀ *X. flavipes* whereas their nymphs prefer nymphs of *L. decolor*. Field evaluation of *X. flavipes* is required for their incorporation into psocid pest management.

## Simple Summary

Managing stored-product psocids with insecticides including phosphine is difficult. The warehouse pirate bug, *Xylocoris flavipes*, is commonly associated with insect pests and mites in storage environments and has potential for use as a biocontrol agent in stored-grain psocid pest management. This study aimed to provide data on the functional responses of adult♀ and nymphs of *X. flavipes* on diets of nymphs, adult♂, and adult♀ of *Liposcelis decolor*, as well as the attack rate, handling time, maximum predation, predation efficiency, per capita consumption rate, and searching efficiency of each type of predator. The results showed that functional responses of adult♀ and nymphs of *X. flavipes* on a diet of *L. decolor* were Holling Type II. Both adult♀ and nymphs of *X. flavipes* preyed on nymphs, adult♂, and adult♀ of *L. decolor* but the consumption rate and searching efficiency of the adult♀ predator were higher than those of the nymphs. The high predation rate of *X. flavipes* indicates positive potential for psocid management.

## 1. Introduction

Psocids (Psocodea: Liposcelididae) are currently regarded as an important taxonomic group of stored-product pests that pose a threat to global food security and safety [1,2,3]. Significant financial losses are incurred when domestic and international trade is restricted and grain shipments for export are rejected due to the presence of psocids [1,4]. Infestation of grains with psocids may cause about 10% weight loss, 54% germ damage, and up to 40% germination failure [5,6,7]. Psocids have become important stored-product pests largely due to their tolerance and resistance to phosphine, the most commonly used and usually effective insecticide against coleopteran and lepidopteran pests of stored products [1,8,9,10]. Psocids are able to inhabit cracks and penetrate the intergranular spaces within the bulk grain and occupy the warm and damp microclimate to feed and damage both the germ and endosperm of infested grain kernels [1,6,7]. Due to phosphine tolerance and resistance, and the prolific nature of psocids and the parthenogenetic reproduction found in certain species, they are able to rapidly colonize new niches and quickly reproduce when conditions are favorable [10,11,12,13].

The use of synthetic chemicals particularly the fumigant, phosphine is the main method for managing stored-grain pests in the United States. However, psocid species including *Liposcelis decolor* (Pearman) can tolerate higher concentrations of phosphine up to 249.76 ppm [10]. Other available control methods include heat disinfestation, dehumidification, and sanitation [7,11,12,13,14]. These methods can be costly, energy-intensive, and less effective due the cryptic nature of psocids.

The potential of natural enemies including predators, parasitoids, antagonists, pathogens, or competitors to suppress pest populations has existed throughout human history [15,16,17]. Several factors including predator traits, prey defenses, hunger, habitat complexity, and competition play critical roles in controlling the rate of predation and influence predator–prey dynamics [18]. For instance, studies using anthocorid predators, including *Orius* spp. and *Antilochus coquebertii*, have found that the sex and age-stage of a predator affect fitness and predation potential [19,20,21].

Biological control can be integrated into stored-product pest management in situations including empty store rooms or warehouse treatment with bioagents, the preventative application of predators and parasitoids to protect packaged or bagged commodities, and the conservation of bulk commodities [22,23,24]. Biological control agents target specific pest species, reducing reliance on broad-spectrum insecticides. This approach is less harmful to humans, environmentally friendly, sustainable, and can serve as an additional method for pest control in a domain where there are limited available insecticides, and insect pests are becoming more resistant to existing synthetic insecticides [10,17]. In Europe, for example, the commercialization and use of beneficial insects for stored-product environments is becoming well established, and biocontrol has proven to be an effective and viable practice in bakeries, wholesale stores, retail shops, mills, and processing companies [23,24]. Commercially available bioagents used against stored-product insects may be parasitoids such as *Trichogramma evanescens* Westwood (Hymenoptera: Trichogrammatidae), *Habrobracon hebetor* (Say) (Hymenoptera: Braconidae), and predators including the predatory mite, *Cheyletus eruditus* (Schrank) (Trombidiformes: Cheyletidae), and the anthocorid *Xylocoris flavipes* (Reuter) (Hemiptera: Anthocoridae). Entomopathogens include *Aspergillus parasiticus* Speare (Eurotiomycetes: Aspergillaceae), *Beauveria bassiana* (Bals.-Criv.) Vuill (Sordariomycetes: Cordycipitaceae), *Isaria fumosoroseus* Wize (Sordariomycetes: Cordycipitaceae), and *Metarhizium anisopliae* (Metchnikoff) (Sordariomycetes: Clavicipitaceae) [23,25,26].

*Xylocoris flavipes*, commonly referred to as the warehouse pirate bug, is one of the known efficient predators of the eggs, larvae, and pupae of small-sized, externally developing stored-product pests [23,27,28]. It is commonly associated with stored-product beetles including *Tribolium castaneum* (Herbst), *T. confusum* Jacqueline du Val, *Lasioderma serricorne* (F.), *Sitophilus zeamais* Motschulsky, and *S. granarius* (L.) and bruchids [29,30,31], as well as moths including *Sitotroga cerealella* (Oliver), *Plodia interpunctella* (Hübner), and *Corcyra cephalonica* Stainton [32,33,34]. *Xylocoris flavipes* has a high capacity to increase in population relative to its prey, and it destroys large numbers of prey when abundant [28]. The warehouse pirate bug has the natural ability to penetrate a grain mass and has been reported in storage facilities worldwide [35,36]. *Xylocoris flavipes* is among several bioagents that have been approved for use against stored-product insect pests in the United States [26].

In recent studies, Danso et al. [17,37] reported that two predatory mites, *C. eruditus* and *C. malaccensis* Oudemans, showed good potential to reduce populations of stored-product psocids. Currently, there is no published information on the predatory characteristics of *X. flavipes* with stored-product psocids as prey. Considerable knowledge about a predator’s foraging behaviors including its functional response is critical for the selection of efficient biocontrol agents [38]. The functional response of a predator defines the numbers of prey it attacks in relation to different prey densities [39,40,41]. Functional response plays a critical role in the dynamics of predator–prey relationships and in practice has been used to improve the ability to predict the potential of predator candidates for biocontrol [42]. Functional response models help to evaluate key parameters including the attack rate—the rate at which a predator searches for and attacks prey—and handling time—the time needed to pursue, subdue, and consume each prey item [40]. These parameters together with the time of predator exposure to prey determine the maximum predation rate—the highest number of prey a predator can consume in a given time period—and the predation efficiency—the number of encounters that resulted in a successful kill of prey [43]. Against this background, the current study evaluated the functional responses of adult♀ and nymphs of *X. flavipes* on diets of different life stages of *L. decolor* (nymphs, adult♂, and adult♀) to assess the potential of this predator for the management of stored-product psocids.

## 2. Materials and Methods

### 2.1. Rearing of Liposcelis decolor

In this study, *Liposcelis decolor* were used as prey for *X. flavipes*. The prey was reared on a psocid diet as described in [17]. *L. decolor* nymphs, adult males (hereafter referred to as adult♂), and adult females (hereafter referred to as adult♀) from the established cultures were selected and used for this study.

### 2.2. Rearing of Xylocoris flavipes

Laboratory stock cultures of *X. flavipes* were initially obtained from Biologische Beratung GmbH, Berlin, Germany. Subsequently, colonies of the warehouse pirate bug were maintained on *L. decolor*. The *L. decolor* were reared on a psocid diet as described in [11,17]. After 4 weeks of incubation, about 50 pairs of *X. flavipes* males and females were introduced into the jars to feed on *L. decolor*. The jars containing both *X. flavipes* and *L. decolor* were placed in plastic boxes as described in [17], which contained saturated NaNO_2_ solution (sodium nitrite, anhydrous, free-flowing, Redi-DriTm, ACS reagent, ≥99%, 746398-2.5 KG, Sigma-Aldrich, Inc., St. Louis, MO, USA) beneath perforated false floors to maintain 63 ± 2% RH. The plastic boxes were then placed inside a growth chamber and maintained at 28 ± 1 °C and a 0:24 (L:D) photoperiod for the *X. flavipes* to multiply and establish. Given that the warehouse pirate bug is cannibalistic, the cultures were frequently monitored and *L. decolor* added biweekly to prevent decline in predator populations because of starvation or conspecific predation. Stock cultures of *X. flavipes* were maintained for at least three generations in the laboratory before they were used for this study.

### 2.3. Experimental Arenas

Experimental arenas consisted of a 5.0 cm diameter basal Petri dish covered by a 5.5 cm diameter lid (forming a total cylindrical surface area of 54.98 cm^2^; the total migration area for a predator in a cylinder of one basal Petri dish and a lid) (50 × 10 mm and 55 × 10 mm Style Polystyrene, Falcon^®^, Becton Dickinson and Company, Franklin Lakes, NJ, USA). The experimental arenas were prepared as described in [17], with 29.99 cm^2^ as the total migration area of prey.

### 2.4. Functional Responses of Adult♀ and Nymphs of X. flavipes

Five- to eight-day-old adult females (hereafter referred to as adult♀) and 3rd to 4th instar nymphs (hereafter referred to as nymphs) of the predator were selected from pure cultures of *X. flavipes*, and they were starved for 24 h prior to being placed in arenas containing their prey, that is, nymphs, adult♂, or adult♀ of *L. decolor* at varying densities. Adult♀ *X. flavipes* of this age were selected because of the preoviposition period (the time between adult emergence and oviposition of the first eggs), which was at least four days. Starvation decreased oviposition, standardized their level of hunger, and initiated a nomadic period [43,44]. Experimental arenas with either adult♀ or nymphs of *X. flavipes* had different densities of the specific prey stage. Prey densities of 2, 6, 15, 30, 40, 50, 60, 70, and 80 were transferred as described in [17]. Each predator type × prey density × prey type combination was replicated six times during the determination of functional responses of *X. flavipes*. Each of the six replications was run separately at different times. Culture jars from which predators and prey were collected and used for each replicate were different (that is, insects in a particular jar or set of jars were used for only one replicate). Each replicate was conducted using the method described in [17]. Thus, combinations of 2-predator stages × 3-prey stages × 9-prey densities were run as a replicate each time, and a total of 6 different runs or 6 replications for each factor level combination was conducted. For each replication set consisting of 54 experimental arenas (2 × 3 × 9), all arenas were arranged randomly in a single plastic box (42 × 29 × 24 cm high) painted black, which contained saturated NaNO_2_ solution beneath perforated false floors to maintain 63 ± 2% RH, and a box was kept inside a growth chamber maintained at 28 ± 1 °C and a 0: 24 (L:D) photoperiod. Arenas were assessed at 24 h intervals to count the number of prey killed by adult♀ and nymphs of *X. flavipes* using the method described in [17]. The number of predators (adult♀ or nymphs of *X. flavipes*) used in the assessment of just one prey stage (each prey stage) for all the 6 replications was a total of 54 (1 prey stage × 9 prey densities × 6 replications). Data on each batch of replicates were collected over 120 h (5 days) and five data sets per adult♀ or nymph of *X. flavipes* were recorded at 24 h intervals. In a nutshell, for each factor level combination (2-predator stages × 3-prey stages × 9-prey densities), there were five 24 h observations. This means the number of 24 h observations in each replicate was 54 × 5, which is 270. The daily average number of cadavers at the end of the fifth consecutive datum (day) was assumed to represent the daily prey consumption rate of adult♀ or nymphs of *X. flavipes* in their confined arenas at a specified prey density. The mean number of prey killed, *Na*, and the searching efficiency, *Na*/*N*, were compared across the three prey stages (nymphs, adult♂, or adult♀) and nine prey densities (2, 6, 15, 30, 40, 50, 60, 70, and 80) for adult♀ and nymphs *X. flavipes*. The experimental setup was a Completely Randomized Design (CRD) replicated 6 different times.

### 2.5. Statistical Analysis

A logistic regression of the proportion of prey consumed (*Na*/*N*) as a function of initial prey density (*N*) was used to determine the shape of the functional response curves of predators (adult♀ or nymphs of *X. flavipes*) to different prey stages (nymphs, adult♂, or adult♀) of *L. decolor*. That is, *Na*/*N* = *exp* (*P*_0_ + *P*_1_ *N* + *P*_2_ *N*^2^ + *P*_3_ *N*^3^)/1 + *exp* (*P*_0_ + *P*_1_ *N* + *P*_2_ *N*^2^ + *P*_3_ *N*^3^), where *Na* is the number of prey consumed and *N* is the initial prey density. *P*_0_, *P*_1_, *P*_2_, and *P*_3_ are the maximum likelihood estimates (MLEs) of the intercept, linear, quadratic, and cubic coefficients, respectively. The signs of *P*_1_ and *P*_2_ were used to determine the type of functional response [45]. The predator displays a Type II functional response when the linear coefficient is significantly negative (*P*_1_ < 0), which indicates that the proportion of prey consumed declines monotonically with the initial prey density. When the linear coefficient is positive (*P*_1_ > 0), and the quadratic coefficient is negative (*P*_2_ < 0), the predator has a Type III functional response [45]. The significantly negative coefficients of *P*_1_ in MLE confirmed a Type II functional response of *X. flavipes* to the different prey stages of *L. decolor*.

Subsequently, Type II functional response curves were fitted for the observed number of prey killed per capita per day using the Holling [39,40] disc equation: *Na* = *aTN*/(1 + *aT_h_N*). In this model, *Na* is the number of prey killed, *N* is the initial density of prey, *T* is the time available for searching during the experiment (*T* = 1 day), and *a* and *T_h_* are the attack rate and the time required to handle prey items, respectively. A linear transformation of Holling’s disc equation 1/*Na =* [(1/*aNT*) *+* (*T_h_*/*T*)] was used to estimate the parameters *a* and *T_h_* using linear regression, where 1/*Na* was regressed on 1/*N*. The reciprocal of the slope of the fitted line by least squares and the time of exposure (*T* = 1 day) multiplied by the y-intercept were the attack rate and the handling time, respectively [46,47].

The mean number of prey killed, *Na*, and the searching efficiency, *Na*/*N*, were compared across the three prey stages (nymphs, adult♂, or adult♀) and nine prey densities (2, 6, 15, 30, 40, 50, 60, 70, and 80) using generalized linear mixed model methods for each of the predator stages (adult♀ and nymphs *X. flavipes*). The model included the main effects, prey stage and prey density, and their interaction for each of the response variables. Least squares means were compared for the appropriate significant effects using the Tukey method. All tests were conducted at the nominal 0.05 level of significance. The 2-predator stages × 3-prey stages × 9-prey densities factor level combination was analyzed using SAS software Version 9.4 (SAS Institute, Cary, NC, USA).

## 3. Results

### 3.1. Functional Response Curves

**Figure 1:**
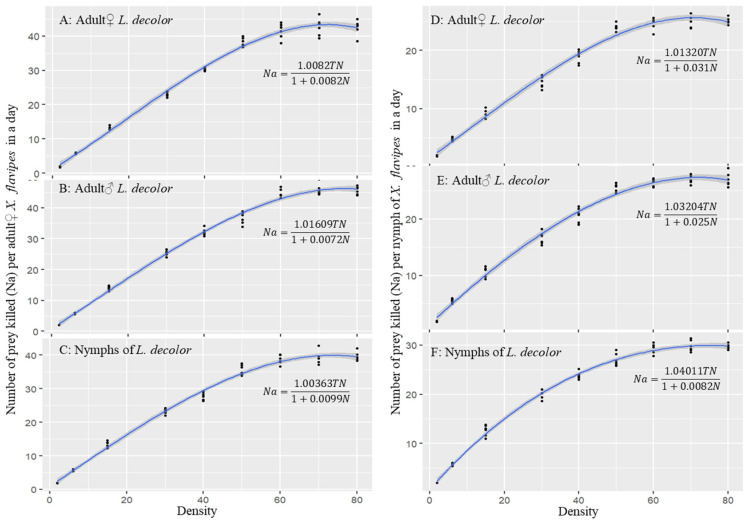
Functional responses of adult♀ and nymphs of *Xylocoris flavipes* on a diet of different life stages (adult♀, adult♂, and nymph) and densities of *Liposcelis decolor*.

The maximum likelihood estimate (MLE) by logistic regression of the proportion of prey consumed (*Na*/*N*) as a function of initial prey density (*N*) per day showed a Type II functional response for *X. flavipes* when on diets of three different life stages of *L. decolor*. Type II functional response is indicated by the significant negative coefficient of the linear parameter (*P*_1_) (Table 1). For both adult♀ and nymphs of *X. flavipes*, there was a significant (*P* < 0.05) negative linear coefficient (*P*_1_ < 0) and positive quadratic coefficient (*P*_2_ > 0) for all three prey stages (Table 1). This indicates that the proportion of prey consumed by either adult♀ or nymphs of *X. flavipes* decreased with increasing prey density for all three prey stages of *L. decolor* (Figure 1A–F).

### 3.2. Holling Type II Functional Response Models for Fitted Curves

A Type II functional response model, a cyrtoid curve rising at a decreasing rate to a plateau with increasing prey density, indicated a significant linear relationship between the number of prey killed and the initial prey density for all prey stages and predators (Table 2 and Figure 1). For both adult♀ and nymphs of *X. flavipes*, strong R^2^ values were observed across the three prey stages. The attack rate was not significantly different (*p* > 0.05) across all the prey stages for adult♀ and nymphs of *X. flavipes* when on a diet of different life stages of *L. decolor*, with an overall average attack rate of 1.019 d^−1^. There were no significant differences (*p* > 0.05) in handling time (*T_h_*) for adult♀ *X. flavipes* when on a diet of different stages of *L. decolor*. Similarly, nymphs of *X. flavipes* did not show any significant differences (*p* > 0.05) in handling time across prey stages (Table 2). However, the estimated average handling time of adult♀ *X. flavipes* (0.008 d, that is, ~11.52 min) was 3 times less or faster than that of the nymphs (0.024 d, that is, ~34.56 min) (Table 2). For the maximum predation rate (*K*) and predation efficiency (*η*), adult♀ or nymphs of *X. flavipes* exhibited no differences (*p* > 0.05) across prey stages. The average maximum predation (121.749 prey d^−1^) and predation efficiency (122.667 prey d^−1^) of adult♀ *X. flavipes* were 2.83 times and 2.77 times, respectively, higher than those of the nymphs, which were 44.001 prey d^−1^ and 44.321 prey d^−1^, respectively (Table 2).

### 3.3. Per Capita Predation and Searching Efficiency of Adult♀ or Nymphs of X. flavipes on a Diet of L. decolor at Different Life Stages and Densities

**Figure 2:**
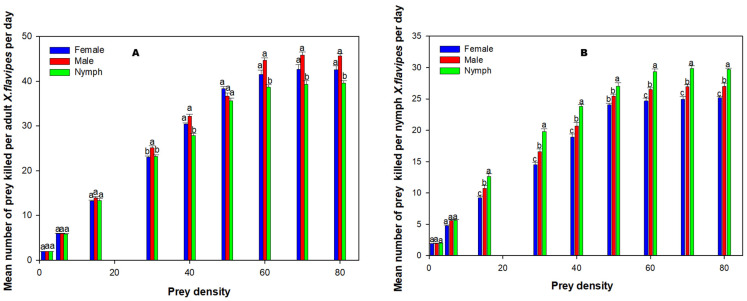
Mean numbers of prey killed (*Na*) per adult♀ of *Xylocoris flavipes* per day (**A**) and nymphs of *Xylocoris flavipes* (**B**) on diets of adult♀, adult♂, and nymph of *L. decolor* at varying densities. Means were analyzed using a two-way analysis of variance at a significance level of α = 0.05, followed by Tukey’s Honestly Significant Difference test for multiple comparison. Significant differences among prey stages for each prey density are denoted with different lowercase letters for each predator.

With the exception of the searching efficiency of adult♀ *X. flavipes*, there were significant interactions (*p* < 0.05) between the prey stage and density in relation to the per capita predation and per capita searching efficiency for adult♀ and nymphs of *X. flavipes* (Table 3). The main effects, prey stage and density, were significant (*p* < 0.05) for both adult♀ and nymphs of *X. flavipes*. At lower prey densities of 2, 6, and 15, adult♀ *X. flavipes* predation across all prey stages was similar; however, as prey density increased, adult♀ *X. flavipes* killed more adult♂ or adult♀ than nymphs of *L. decolor* (Figure 2). On the contrary, more nymphs of *L. decolor* were killed by nymphs of *X. flavipes* at higher prey densities (Figure 2). Searching efficiency of both adult♀ and nymphs of *X. flavipes* was higher at lower prey densities across all prey stages (Table 4). Adult♀ *X. flavipes* were generally more efficient for adult♂ and adult♀ than nymphs of *L. decolor*. On the contrary, the searching efficiency of nymphs of *X. flavipes* was higher on the nymphs of *L. decolor* than the adult prey stages (Table 4).

## 4. Discussion

The warehouse pirate bug, *X. flavipes* is commonly associated with stored-product beetles and moths worldwide [34,48,49]. However, there is little to no information on its predatory activities and capacity to manage stored-product psocids. From the current study, both adult♀ and nymphs of *X. flavipes* prey on mobile life stages (nymphs, adult♂, and adult♀) of *L. decolor*. Many previous studies have found that the warehouse pirate bug is an efficient predator of the eggs, larvae, and pupae of externally developing stored-product pests in different storage systems and climatic conditions [29,35,49]. Generally, predators have a higher foraging rate when there is more prey, and the relationship between the foraging rate and prey density is known as the functional response [39,40,50]. Functional response of a predator influences pest population dynamics and is useful for determining the density at which a targeted pest would escape control. Functional response is also a key component in assessing biocontrol agents [43,51]. The type of functional response of adult♀ and nymphs of *X. flavipes* when on diets of *L. decolor* observed in this study was Holling Type II. This Type II functional response indicates that predators consume more prey with increasing prey availability until a plateau is reached where consumption stabilizes and becomes constant [17,52,53]. Type II functional response exhibited by *X. flavipes* in this study is consistent with previous studies on *X. flavipes* and many other predatory insects used in biocontrol [17,35,54,55]. Insect predators can exhibit either a Type II or Type III or both functional responses. Nonetheless, Type II is most common in many predators released as biocontrol agents [56]. The increased predation at high prey densities observed in the present study can be attributed to high encounter rates due to the confined space, as well as disturbance of predators during feeding, causing them to kill prey wastefully or defensively [17,57]. Density-dependent wasteful killing where more prey is partially consumed is known to be an adaptive foraging strategy restricted in time and space, rather than an evolved predatory behavior [57,58]. This suggests that *X. flavipes* has the potential to exert more predation pressure on *L. decolor* to substantially suppress its population. According to Trubl et al. [57], wasteful killing may be an aggression strategy used by predators to suppress thresholds of pests below an economic injury level.

The major factors that limit a predator’s maximum predation include the total time of exposure of the predator to prey, predator’s attack rate, and handling time [53]. The current study showed that adult♀ and nymphs of *X. flavipes* had similar attack rates on the mobile life stages of *L. decolor*, indicating that both life stages of the predator are equally capable of locating and initiating an attack on *L. decolor*. However, the handling time, defined as the time a predator requires to pursue, subdue, consume prey, clean and rest before a new search, was shorter in the adult♀ *X. flavipes* than the nymphs of *X. flavipes* for all prey stages. In a related study, Danso et al. [17] observed that *C. eruditus* had a shorter handling time compared with *C. malaccensis* when preying on *L. decolor*. Previous studies have shown that handling time can vary significantly between predators and across prey stages and influence the nature of the response of predators to different prey stages [17]. With shorter handling times, the predation efficiency of adult♀ of *X. flavipes* was higher compared to the nymphs of *X. flavipes*—the ratio of the attack rate to the handling time provides a good indicator of predation efficiency—encounters that result in a successful kill [43,59]. The ability of adult♀ *X. flavipes* to process *L. decolor* more quickly and consume larger numbers of prey suggests that they might be more effective for faster control of high pest population densities than the nymphs. According to Danso et al. [17], *C. eruditus* had a shorter handling time when preying on *L. decolor*, which implied that it would be more efficient for managing psocids in a storage environment compared to *C. malaccensis* with a longer handling time. However, the attack rate and handling time of predators are influenced by an interplay of complex factors including predator and prey behavior, prey defense mechanisms, and environmental conditions [59]. Therefore, although laboratory estimates of a predator’s functional response parameters provide valuable insights into predator–prey dynamics, they may overestimate predatory capacity; hence, extrapolating to storage conditions in the field should be performed cautiously [60].

Based on this study, differential prey stage preferences were observed between adult♀ and nymphs of *X. flavipes* at high prey densities. Adult♀ *X. flavipes* killed more adult stages of *L. decolor* than the nymphs of the prey whereas nymphs of *X. flavipes* preferred the nymphs of *L. decolor* compared with the adult stages. This observation differs from that reported by Danso et al. [17] for *C. eruditus* and *C. malaccensis*, where both predator species killed more nymphs of *L. decolor* than adult♂ and adult♀. It can be explained that because adult♀ *X. flavipes* are larger and more developed, they are able to handle larger, more nutritionally valuable adult *L. decolor* to meet their high energy demand for egg production and bodily maintenance [35,44]. A study by Lecato and Davis [27] found that *X. flavipes* preferred large-sized larvae of small prey species and small-sized larvae when prey species were large. Another possible explanation is that at high prey densities, predators sometimes exhibit a trade-off between the quantity and quality of their prey and they are assumed to maximize energy intake rates by opting for quality prey [61]. In a related functional response study, Donnelly and Philips [35] found that female *X. flavipes* killed more prey than males, indicating that factors including sex also influence predation. For nymphs of *X. flavipes*, they are less developed and may be better adapted to handle *L. decolor* nymphs, which are smaller with less structural defenses to resist attack [17,35,44]. Nonetheless, de Oliveira et al. [62] found no difference in predation capacity between nymphs and adults of *Xylocoris sordidus* (Hemiptera: Anthocoridae) when feeding on the peanut thrips, *Enneothrips enigmaticus* (Thysanoptera: Thrypidae). The observed form of resource partitioning between adult♀ and nymphs of *X. flavipes* may have significant implications for the release time and effectiveness due to concurrent disruption of both the reproductive units and the immature stages of *L. decolor*. Additionally, by exploiting different components of the prey population, competition between the predator life stages is reduced and ensures more stable and sustainable pest suppression [63,64].

In the present study, the searching efficiency of adult♀ and nymphs of *X. flavipes* decreased as the prey density increased, which is consistent with previous studies on predatory mites that showed a Type II functional response when feeding on *L. decolor* [17]. Several variables including the searching arena and spatial complexity, competitors, and environmental conditions impact the searching efficiency of predators [65]. The observed decrease in searching efficiency with increasing prey density indicates that the deployment of *X. flavipes* may be most effective at low to moderate *L. decolor* population densities.

## 5. Conclusions

The current study shows that *Xylocoris flavipes* has potential to manage all mobile stages of *Liposcelis decolor*. The observed preferences of adult♀ *X. flavipes* for adult *L decolor*, and nymphs of *X. flavipes* for nymphs of *L. decolor*, present opportunities for the targeted release of *X. flavipes* for the optimal control of psocids. To enhance the incorporation of *X. flavipes* into psocid IPM systems, further studies under field conditions and assessment of its compatibility with other stored-product pest management methods should be conducted.

## Figures and Tables

**Table 1:** Maximum likelihood estimates from logistic regressions of the proportions of *Liposcelis decolor* ♀, ♂, and nymphs consumed by adult♀ or nymphs of *Xylocoris flavipes* as a function of initial prey density.

Predator	Prey Stage	Parameter	Estimate	Standard Error	Chi-Square (χ^2^)	*p*-Value
Adult♀ *X. flavipes*	Female	Intercept (*P*_0_)	3.5969	0.6847	27.6007	<0.0001
		Linear (*P*_1_)	−0.1303	0.0470	7.6938	0.0055
		Quadratic (*P*_2_)	0.00237	0.00101	5.5262	0.0187
		Cubic (*P*_3_)	−0.00002	0.0000067	5.8132	0.0159
	Male	Intercept (*P*_0_)	4.8241	0.9347	26.6389	<0.0001
		Linear (*P*_1_)	−0.1806	0.0604	8.9428	0.0028
		Quadratic (*P*_2_)	0.00310	0.00123	6.3143	0.0120
		Cubic (*P*_3_)	−0.00002	0.0000079	6.0423	0.0140
	Nymph	Intercept (*P*_0_)	3.7547	0.6971	29.0105	<0.0001
		Linear (*P*_1_)	−0.1409	0.0473	8.8845	0.0029
		Quadratic (*P*_2_)	0.00237	0.00101	5.5390	0.0186
		Cubic (*P*_3_)	−0.00001	0.0000066	5.1165	0.0237
Nymph *X. flavipes*	Female	Intercept (*P*_0_)	1.8712	0.4185	19.9931	<0.0001
		Linear (*P*_1_)	−0.1111	0.0323	11.8468	0.0006
		Quadratic (*P*_2_)	0.00208	0.000752	7.6244	0.0058
		Cubic (*P*_3_)	−0.00001	0.0000053	6.9291	0.0085
	Male	Intercept (*P*_0_)	2.7667	0.5007	30.5352	<0.0001
		Linear (*P*_1_)	−0.1438	0.0366	15.4136	<0.0001
		Quadratic (*P*_2_)	0.00253	0.000823	9.4421	0.0021
		Cubic (*P*_3_)	−0.00002	0.0000059	7.9996	0.0047
	Nymph	Intercept (*P*_0_)	3.5212	0.6190	32.3601	<0.0001
		Linear (*P*_1_)	−0.1513	0.0428	12.4832	0.0004
		Quadratic (*P*_2_)	0.00235	0.000925	6.4674	0.0110
		Cubic (*P*_3_)	−0.00001	0.0000062	4.9282	0.0264

**Table 2:** Parameter estimates for Holling Type II functional responses of adult♀ or nymphs of *Xylocoris flavipes* on diets of different life stages and densities of *Liposcelis decolor*.

Predator	Prey Stage	*a* (d^−1^)	*T_h_* (d)	*K* (Prey d^−1^)	*η* (Prey d^−1^)	*p*-Value	R^2^ [n]
Adult♀ *X. flavipes*	Female	1.010 a	0.008 a	122.294 a	122.395 a	<0.0001	0.998 (54)
	Male	1.016 a	0.007 a	141.985 a	144.269 a	<0.0001	0.999 (54)
	Nymph	1.004 a	0.009 a	100.969 a	101.336 a	<0.0001	0.997 (54)
Nymph *X. flavipes*	Female	1.013 a	0.031 b	32.591 b	33.022 b	<0.0001	0.993 (54)
	Male	1.032 a	0.024 b	41.943 b	43.287 b	<0.0001	0.996 (54)
	Nymph	1.040 a	0.018 b	54.469 b	56.654 b	<0.0001	0.999 (54)

Parameterc estimates were predicted by a linear transformation of Holling’s disc equation: 1/*Na* = [(1/*aNT*) + (*Th*/*T*)]. Instantaneous attack rate (*a*); Handling time (*T_h_*); Total time available for searching (*T*) = 1 d; Maximum predation rate (*K*) = (*T*/*T_h_*); Predation efficiency (*η*) = *a*/*T_h_*. Significant differences within columns are denoted with different lowercase letters.

**Table 3:** Summary of the tests for the main effects of the prey stage (PS) and prey density (N) of *Liposcelis decolor* on the numbers of prey killed (*Na*) and searching efficiency (*Na*/*N*) of adult♀ and nymphs *Xylocoris flavipes*.

Predator	Variable	Source	Df	*F*	*p*-Value
Adult♀ *X. flavipes*	*Na*	PS	2, 135	71.37	<0.0001
		N	8, 135	2797.49	<0.0001
		PS*N	16, 135	7.80	<0.0001
	*Na*/*N*	PS	2, 135	11.50	<0.0001
		N	8, 135	149.92	<0.0001
		PS*N	16, 135	1.30	<0.2042
Nymph of *X. flavipes*	*Na*	PS	2, 135	232.05	<0.0001
		N	8, 135	2447.86	<0.0001
		PS*N	16, 135	7.72	<0.0001
	*Na*/*N*	PS	2, 135	38.07	<0.0001
		N	8, 135	170.92	<0.0001
		PS*N	16, 135	5.91	<0.0001

**Table 4:** Per capita searching efficiency (*Na*/*N*) (mean ± SE) of adult♀ and nymphs of *Xylocoris flavipes* on a diet of *Liposcelis decolor* of different developmental stages and varying densities.

Predator	Prey Density (*N*)	Searching Efficiency (*N_a_*/*N*)		
		Prey Stage		
		Female	Male	Nymph
Adult♀ *X. flavipes*	2	0.99 ± 0.00 A	0.99 ± 0.00 A	0.99 ± 0.00 A
	6	0.99 ± 0.00 A	0.99 ± 0.00 A	0.99 ± 0.00 A
	15	0.88 ± 0.01 bB	0.94 ± 0.01 aA	0.90 ± 0.01 bB
	30	0.76 ± 0.02 bC	0.84 ± 0.02 aB	0.77 ± 0.02 bC
	40	0.76 ± 0.02 aC	0.80 ± 0.02 aB	0.69 ± 0.02 bC
	50	0.76 ± 0.01 aC	0.73 ± 0.02 aC	0.71 ± 0.02 bC
	60	0.69 ± 0.02 bD	0.74 ± 0.03 aC	0.64 ± 0.02 cD
	70	0.61 ± 0.02 aE	0.65 ± 0.02 aD	0.56 ± 0.02 bD
	80	0.53 ± 0.02 bF	0.60 ± 0.02 aD	0.49 ± 0.03 cE
	2	0.99 ± 0.00 A	0.99 ± 0.00 A	0.99 ± 0.00 A
Nymph of *X. flavipes*	6	0.80 ± 0.02 bB	0.97 ± 0.01 aA	0.97 ± 0.01 aA
	15	0.62 ± 0.02 cC	0.72 ± 0.02 bB	0.85 ± 0.08 aB
	30	0.48 ± 0.02 cD	0.55 ± 0.02 bC	0.66 ± 0.02 aC
	40	0.47 ± 0.02 bD	0.52 ± 0.02 bC	0.59 ± 0.02 aD
	50	0.48 ± 0.01 bD	0.51 ± 0.03 aC	0.54 ± 0.02 aD
	60	0.41 ± 0.02 cE	0.44 ± 0.01 bD	0.49 ± 0.02 aE
	70	0.36 ± 0.02 bE	0.39 ± 0.02 aD	0.43 ± 0.01 aF
	80	0.32 ± 0.01 bF	0.34 ± 0.01 aE	0.37 ± 0.02 aG

Significant differences among prey stages for each prey density are denoted with different lowercase letters (within the same row) for each predator, and differences among prey densities for each prey stage are denoted by different uppercase letters (within a column) for each predator (*p* < 0.05, Tukey).

## Data Availability

The original contributions presented in this study are included in the article. Further inquiries can be directed to the corresponding author.

## References

[1] Nayak M.K., Collins P.J., Throne J.E., Wang J.J. (2014). Biology and management of psocids infesting stored products. Annu. Rev. Entomol..

[2] Stejskal V., Hubert J., Aulicky R., Kučerová Z. (2015). Overview of present and past and pest-associated risks in stored food and feed products: European perspective. J. Stored Prod. Res..

[3] Wei D.D., He W., Miao Z.Q., Tu T.Q., Wang L., Dou W., Wang J.J. (2020). Characterization of esterase genes involving malathion detoxification and establishment of an RNA interference method in *Liposcelis bostrychophila*. Front. Physiol..

[4] Ahmedani M.S., Shagufta N., Aslam M., Hussnain S.A. (2010). Psocid: A new risk for global food security and safety. Appl. Entomol. Zool..

[5] Stejskal V., Douda O., Zouhar M., Throne J.E., Opit G.P., Flinn P.W., Lorini I., Bacaltchuk B., Beckel H., Deckers E., Sundfeld E., Dos Santos J.P., Biagi J.D., Celaro J.C., Faroni L.R.D.A., Bortolini L. (2006). Seasonal distribution of psocids in stored wheat. Proceedings of the 9th International Working Conference on Stored-Product Protection.

[6] Athanassiou C.G., Arthur F.H., Throne J.E., Opit G.P., Hasan M.M., Aikins M.J., Phillips T.W., Kavallieratos N.G., Carvalho O.M., Fields P.G., Adler C.S., Arthur F.H., Athanassiou C.G., Campbell J.F., Fleurat-Lessard F., Flinn P.W., Hodges R.J., Isikber A.A. (2010). Efficacy of insecticides for control of stored-product psocid. Proceedings of the 10th International Working Conference on Stored Product Protection.

[7] Gautam S.G., Opit G.P., Giles K.L., Adam B. (2013). Weight loss and germination failure caused by psocids in different wheat varieties. J. Econ. Entomol..

[8] Athanassiou C.G., Rumbos C.I., Athanassiou C.G., Arthur F.H. (2018). Emerging pests in durable stored products. Recent Advances in Stored Product Protection.

[9] Diaz-Montano J., Campbell J.F., Phillips T.W., Throne J.E. (2018). Evaluation of light attraction for the stored-product psocids, *Liposcelis entomophila*, *Liposcelis paeta*, and *Liposcelis brunnea*. J. Econ. Entomol..

[10] Danso J.K., Opit G.P., Noden B.H., Giles K.L. (2022). Estimating discriminating doses of phosphine for adults of eight species of psocids of genera *Liposcelis* (Psocodea: Liposcelididae) and *Lepinotus* (Psocodea: Trogiidae). J. Stored Prod. Res..

[11] Opit G.P., Throne J.E. (2008). Population growth and development of the psocid *Lepinotus reticulatus* at constant temperatures and relative humidities. J. Econ. Entomol..

[12] Athanassiou C.G., Kavallieratos N.G., Throne J.E., Nakas C.T. (2014). Competition among species of stored product psocids in stored grains (Psocoptera). PloS ONE.

[13] Ocran A.F., Opit G.P., Noden B.H., Arthur F.H., Kard B.M. (2021). Effects of dehumidification on the survivorship of four psocid species. J. Econ. Entomol..

[14] Phillips T.W., Throne J.E. (2010). Biorational approaches to managing stored product insects. Ann. Rev. Entomol..

[15] Smith H.S. (1919). On some phases of insect control by the biological method 1. J. Econ. Entomol..

[16] Huffaker C.B., Messenger P.S. (1976). Theory and Practice of Biological Control.

[17] Danso J.K., Opit G.P., Goad C.L., Noden B.H., Giles K.L. (2023). Functional responses of predatory mites, *Cheyletus eruditus* (Schrank) and *Cheyletus malaccensis* Oudemans (Trombidiformes: Cheyletidae) to *Liposcelis decolor* (Pearman) (Psocodea: Liposcelididae). J. Stored Prod. Res..

[18] DeLong J.P. (2021). Predator Ecology: Evolutionary Ecology of the Functional Response.

[19] Ali S., Li S., Jaleel W., Khan M.M., Wang J., Zhou X. (2020). Using a two-sex life table tool to calculate the fitness of *Orius strigicollis* as a predator of *Pectinophora gossypiella*. Insects.

[20] Ali S., Zhu Q., Jaleel W., Rehman S.U., Rasheed M.A., Khan M.M., Islam Y., Hafeez M., Zhou X. (2020). Determination of fitness traits of *Orius strigicollis* Poppius (Hemiptera: Anthocoridae) on *Pectinophora gossypiella* (Lepidoptera: Gelechiidae) using two-sex life table analysis. PeerJ.

[21] Sarmad M., Jaleel W., Zaka S.M., Saeed Q., Azher F., Rabbani M.K., Ullah R.M.K. (2020). Fitness and predating potential of *Antilochus coquebertii* (Hemiptera: Pyrrhocoridae): A predator of the red cotton bug (*Dysdercus koenigii*). J. Kans. Entomol. Soc..

[22] Hanson L.S. (2007). Potential for widespread application of biolocal control of stored-product pests–the European perspective. J. Stored Prod. Res..

[23] Schöller M.E., Flinn P.W., Grieshop M.J., Zd’árková E., Heaps J.W. (2006). Biological control of stored product pests. Insect Management for Food Storage and Processing.

[24] Prozell S., Schöller M., Credland P.F., Armitage D.M., Bell C.H., Cogan P.M., Highley E. Five years of biological control of stored-product moths in Germany. Advances in Stored Product Protection, Proceedings of the 8th International Working Conference on Stored Product Protection, 22–26 July 2002, York, UK.

[25] Ždárková E., Horák E. (1990). Preventive biological control of stored food mites in empty stores using *Cheyletus eruditus* (Schrank). Crop Prot..

[26] Hagstrum D.W., Subramanyam B. (2006). Fundamentals of Stored-Product Entomology.

[27] LeCato G.L., Davis R. (1973). Preferences of the predator *Xylocoris flavipes* (Hemiptera: Anthocoridae) for species and instars of stored products insects. Fla. Entomol..

[28] Arbogast R.T. (1979). Cannibalism in *Xylocoris flavipes* (Reuter) (Hemiptera: Anthocoridae), a predator of stored-product insects. Entomol. Exp. Appl..

[29] Press J.W., Flaherty B.R., Arbogast R.T. (1975). Control of the red flour beetle, *Tribolium castaneum*, in a warehouse by a predacious bug, *Xylocoris flavipes*. J. Ga. Entomol. Soc..

[30] Schöller M., Prozell S. (2011). Potential of *Xylocoris flavipes* (Hemiptera: Anthocoridae) to control *Tribolium confusum* (Coleoptera:Tenebrionidae) in Central Europe. IOBC/WPRS Bull..

[31] Berger A., Degenkolb T., Vilcinskas A., Schöller M. (2017). Evaluating the combination of a parasitoid and a predator for biological control of seed beetles (Chrysomelidae: Bruchinae) in stored beans. J. Stored Prod. Res..

[32] Reichmuth C., Schöller M., Ulrichs C. (2007). Stored Product Pests in Grain. Morphology, Biology, Damage, and Control.

[33] Rabinder K., Singh V.J. (2011). Role of *Blaptostetus pallescens* Poppius and *Xylocoris flavipes* (Reuter) in the suppression of *Corcyra cephalonica* Stainton in stored rice grain. J. Biol. Ctr..

[34] Suma P.M., Amante S., Bella A., Pergola L., Russo A. (2013). Stored-product insects natural enemies in wheat industry in Sicily. IOBC-WPRS Bull..

[35] Donnelly B.E., Phillips T.W. (2001). Functional response of *Xylocoris flavipes* (Hemiptera: Anthocoridae) effects of prey species and habitat. Environ. Entomol..

[36] Basumatary M.M., Patgiri P., Handique G. (2013). First report of warehouse pirate bug (*Xylocoris flavipes* (Reuter) (Hemiptera: Anthocoridae) on stored paddy from north-east India. Insect Environ..

[37] Danso J.K., Opit G.P., Giles K.L., Noden B.H. (2023). Ecological interactions of predatory mites, *Cheyletus eruditus* (Schrank) (Trombidiformes: Cheyletidae) and *Cheyletus malaccensis* Oudemans, and prey, *Liposcelis decolor* (Pearman) (Psocodea: Liposcelididae), under different thermo-hygrometric regimes. Insects.

[38] Fathipour Y., Maleknia B., Omkar (2016). Mite predators. Ecofriendly Pest Management for Food Security.

[39] Holling C.S. (1959). The components of predation as revealed by a study of small mammal predation of the European pine sawfly. Can. Entomol..

[40] Holling C.S. (1959). Some characteristics of simple types of predation and parasitism. Can. Entomol..

[41] Rahman V.J., Babu A., Roobakkumar A., Perumalsamy K. (2012). Functional and numerical responses of the predatory mite, *Neoseiulus longispinosus* to the red spider mites, *Oligonychus coffeae*, infesting tea. J. Insect Sci..

[42] Sepúlveda F., Carrillo R. (2008). Functional response of the predatory mite *Chileseius camposi* (Acarina: Phytoseiidae) on densities of its prey, *Panonychus ulmi* (Acarina: Tetranychidae). Rev. Biol. Trop..

[43] Opit G.P., Roitberg B., Gillespie D.R. (1997). The functional response and prey preference of *Feltiella acarisuga* (Vollot) (Diptera: Cecidomiidae) for two of its prey: Male and female two-spotted spider mites, *Tetranychus urticae* Koch (Acari: Tetranychiidae). Can. Entomol..

[44] Kucerova Z. (2002). Weight loss of wheat grains caused by psocid infestation (*Liposcelis bostrychophila*: Liposcelididae: Psocoptera). Plt. Proct. Sci..

[45] Juliano S., Scheiner S.M., Gurevitch J. (2001). Nonlinear curve fitting: Predation and functional response curves. Design and Analysis of Ecological Experiments.

[46] Lividahl J.P., Stiven A.E. (1983). Statistical difficulties in the analysis of predation functional response data. Can. Entomol..

[47] Yao H., Zheng W., Tario K., Zhang H. (2014). Functional and numerical responses of three species of predatory Phytoseiid mites (Acari: Phytoseiidae) to *Thrips flavidulus* (Thysanoptera: Thripidae). Neotrop. Entomol..

[48] Loko Y.L., Dansi A., Tamo M., Bokonon-Gantaa H., Assogba P., Dansi M., Vodouhe’ R., Akoegninou A., Sanni A. (2013). Storage insects on yam chips and their traditional management in Northern Benin. Sci. World J..

[49] Adarkwah C., Obeng-Ofori D., Opuni-Frimpong E., Ulrichs C., Schöller M. (2018). Predator-parasitoid-host interaction: Biological control of *Rhyzopertha dominica* and *Sitophilus oryzae* by a combination of *Xylocoris flavipes* and *Theocolax elegans* in stored cereals. Entomol. Exp. Appl..

[50] Solomon M.E. (1949). The natural control of animal populations. J. Anim. Ecol..

[51] Lester P.J., Harmsen R. (2002). Functional and numerical responses do not always indicate the most effective predator for biological control: An analysis of two predators in a two-prey system. J. Appl. Ecol..

[52] Holling C.S. (1961). Principles of insect predation. Ann. Rev. Entomol..

[53] Hassel M.P. (1978). Dynamics of Arthropod Predator-Prey Systems.

[54] Sing S.E., Arbogast R.T. (2008). Optimal *Xylocoris flavipes* (Reuter) (Hemiptera: Anthocoridae) density and time of introduction for suppression of Bruchid progeny in stored legumes. Environ. Entomol..

[55] Schoeller E.N., Hogan J., McKenzie C.L., Osborne L.S. (2024). Functional response of *Franklinothrips vespiformis* (Thysanoptera: Aeolothripidae) to eggs and nymphs of *Bemisia tabaci* (Hemiptera: Aleyrodidae). J. Insect Sci..

[56] Cedola C.V., Sanchez N.L., Lijesthrom G. (2001). Effect of tomato leaf hairiness on functional and numerical response of *Neoseiulus californicus* (Acari: Phytoseiidae). Exp. Appl. Acarol..

[57] Trubl P., Blackmore V., Johnson J.C. (2011). Wasteful killing in urban black widows: Gluttony in response to food abundance. Ethology.

[58] Lounibos L.P., Makhni S., Alto B.W., Kesavaraju B. (2008). Surplus killing by predatory larvae of *Corethrella appendiculata*: Prepupal timing and site-specific attack on mosquito prey. J. Insect Behav..

[59] Bazgir F., Shakaram J., Jafari S. (2020). Functional response of the predatory mite *Amblyseius sweirskii* (Acari: Phytoseiidae) to *Eotetranychus frosti* (Tetranychidae) and *Cenopalpus irani* (Tenuipalpidae). Acarologie.

[60] Zhu P., Fan Y., Mo W., Xin T., Xia B., Zou Z. (2019). Functional response of adult *Cheyletus malaccensis* (Acari: Cheyletidae) to different developmental stages of *Aleuroglyphus ovatus* (Acari: Acaridae). J. Stored Prod. Res..

[61] Bijleveld A.I., MacCurdy R.B., Chan Y., Penning E., Gabrielson R.M., Cluderay J., Spaulding E.L., Dekinga A., Holthuijsen S., ten Horn J. (2016). Understanding spatial distributions: Negative density-dependence in prey causes predators to trade-off prey quantity with quality. Proc. R. Soc. B.

[62] de Oliveira S.J., Nascimento V.F., de Lacerda L.B., de Souza J.M., Ramalho D.M., Izidro Y.E., De Bortoli S.A. (2024). Predator–Prey Interaction Between *Xylocoris sordidus* (Hemiptera: Anthocoridae) and *Enneothrips enigmaticus* (Thysanoptera: Thripidae). Neotrop. Entomol..

[63] Polis G.A., Myers C.A. (1989). The ecology and evolution of intra guild predation: Potential competitors that eat each other. Ann. Rev. Ecol. Syst..

[64] Pekas A., Tena A., Harvey J.A., Garcia-Marí F., Frago E. (2016). Host size and spatiotemporal patterns mediate the coexistence of specialist parasitoids. Ecology.

[65] Gitonga L.M., Overholt W.A., Löhr B., Magambo J.K., Mueke J.M. (2002). Functional response of *Orius albidipennis* (Hemiptera: Anthocoridae) to *Megalurothrips sjostedti* (Thysanoptera: Thripidae). Biol. Cont..

